# Gastrointestinal stromal tumors (GISTs) with remarkable cystic change: a specific subtype of GISTs with relatively indolent behaviors and favorable prognoses

**DOI:** 10.1007/s00432-019-02853-y

**Published:** 2019-03-28

**Authors:** Anwei Xue, Wei Yuan, Xiaodong Gao, Yong Fang, Ping Shu, Chen Xu, He Li, Yifang Xu, Qi Song, Yingyong Hou, Kuntang Shen

**Affiliations:** 1 Department of General Surgery, Zhongshan Hospital, Fudan University, NO. 180 Fenglin Road, Shanghai, 200032 China; 2 Department of Pathology, Zhongshan Hospital, Fudan University, NO. 180 Fenglin Road, Shanghai, 200032 China

**Keywords:** Gastrointestinal stromal tumors, Cystic change, Prognosis

## Abstract

**Purpose:**

Gastrointestinal stromal tumors (GISTs) are typically solid neoplasms with small cystic change detected occasionally but in rare instances may present predominantly as cystic lesions. The histopathologic features and prognoses of cystic GISTs (cGISTs) are poorly understood.

**Methods:**

We herein reviewed 20 cGISTs resected or consulted in our institution from January 1, 2003 to December 31, 2014.

**Results:**

Of the 20 patients included, the mean age was 61 years and the male-to-female ratio was 9:11. The original locations were the stomach (*n* = 10, 50%), the small intestine (*n* = 9, 45%) and the omentum (*n* = 1, 5%). Indistinct diagnosis or misdiagnosis was established in 15 cases based only on preoperative radiology. Grossly, the cystic component made up the bulk of masses and was filled by dark bloody fluid and necrotic debris in 18 cases. Microscopically, cyst wall was composed of neoplastic spindle (*n* = 14, 70%)/epithelioid cells (*n* = 6, 30%) and collagenous fiber, with necrotic debris and granulation tissue lining on the inner surface. cGISTs resembled their solid counterparts in terms of morphology and immunohistology but demonstrated fewer malignant parameters. *c-kit* or *PDGFRα* mutations were detected in eleven cases with the remaining being wild type for these two mutations. Although classified as intermediate or high (3 and 17, respectively) risk of recurrence according to modified National Institute of Health criterion, most patients with cGISTs experienced long-term recurrence-free survival without adjuvant imatinib.

**Conclusions:**

Cystic GISTs is a relatively indolent subset of GISTs with favorable prognoses and adjuvant imatinib should be a prudent consideration.

## Introduction

Gastrointestinal stromal tumors (GISTs) represent the most common mesenchymal tumors arising from the digestive tract, with an estimated annual incidence of 10–20 per million individuals in western countries (Corless 2014). The majority of GISTs are located in the stomach (60–70%), small intestine (25–35%) and duodenum (5%) (Miettinen and Lasota 2006). Immunohistochemical and ultramicrostructural findings indicate that GISTs may originate from the intestinal cells of Cajal, which serve as pacemakers for peristaltic contractions (Kindblom et al. 1998). About 85–95% of GISTs harbor activating mutations in *c-kit* or *PDGFRα* that drives the pathogenesis and progression of the disease (Fletcher et al. 2002; Heinrich et al. 2003). Imatinib mesylate, which targets KIT and PDGFRα, has emerged as an effective therapeutic alternative for advanced GISTs while surgical resection remains the mainstay treatment for resectable ones (von Mehren et al. 2018).

GISTs are typically solid, sometimes with small cystic area developed, but rarely manifest as predominant cystic neoplasms. To date, reports of this uncommon form of GISTs have comprised mostly case reports focusing mainly on its clinical and radiographic features (Hamza et al. 2016; Okano et al. 2015; Shaikh et al. 2015; Sun et al. 2016; Takahashi et al. 2010; Wang et al. 2017; Zhu et al. 2014). There is very limited information in the literature relating to the pathologic features and prognoses of GISTs undergoing extensive cystic change. To better elucidate the characteristics of such lesions, we herein undertook this study of the clinical and morphologic features, the prognoses, and the mutational status of 20 affected patients.

## Materials and methods

Study approval was obtained from the institutional review board at Zhongshan Hospital, Fudan University. Surgical pathology database and consultation files of our hospital were queried for GISTs with cystic change from January 1, 2003 to December 31, 2014. The diagnosis of “cystic gastrointestinal stromal tumors (cGISTs)” was established if the proportion of cystic component was larger than 75% and cyst wall was relatively regular by corresponding gross reports and/or preoperative radiology reports. A total of 20 cases were retrieved, 10 from the surgical pathology database and 10 from the consultation files. Patient variables included age, sex, symptoms on presentation, preoperative radiology and medical history.

Gross pathology reports were assessed for location, size, whether unilocular or multilocular, cystic fluid and septa thickness of the tumor. Hematoxylin and eosin-stained slides or corresponding scanned photographs were reviewed by two experienced pathologists for cellular type (spindle, epithelioid or mixed), cellularity, nuclear atypia, mitotic activity (number of mitoses per 50 high power fields). Lymph node metastasis, vascular, fat, nerve or mucosal infiltration, mitoses ≥ 10/50 HPF, muscularis propria infiltration, coagulative necrosis, perivascular pattern and severe nuclear atypia were considered as malignant biological behaviors and evaluated in all cases (Hou et al. 2009a, b).

Immunohistochemistry of CD117, desmin and CD34 were performed in all cases using formalin-fixed paraffin-embedded tissue sectioned at 4 µm while S-100, SMA and DOG-1 immunostain were available for analysis in a varying number of cases. Selected mutation hotspots in *c-kit* exons 9, 11, 13 and 17 as well as *PDGFRα* exons 12, 14 and 18 were examined using polymerase chain reaction (PCR) and Sanger sequencing.

To compare the prognostic factors and outcomes between solid and cystic GISTs, our surgical pathology database was searched for solid GISTs of comparable external size and resected during the same period. Finally, 200 counterparts were identified and clinicopathological characteristics were compared using Chi square test or non-parametric test. Recurrence-free survival (RFS) was defined as the period between surgical resection and radiologic evidence of recurrence. Survival curves were computed by Kaplan–Meier product limit method with intergroup difference compared by log-rank test. All the tests were two-sided and statistical significance was defined as a *P* value < 0.05.

## Results

### Clinical features

**Fig. 1:**
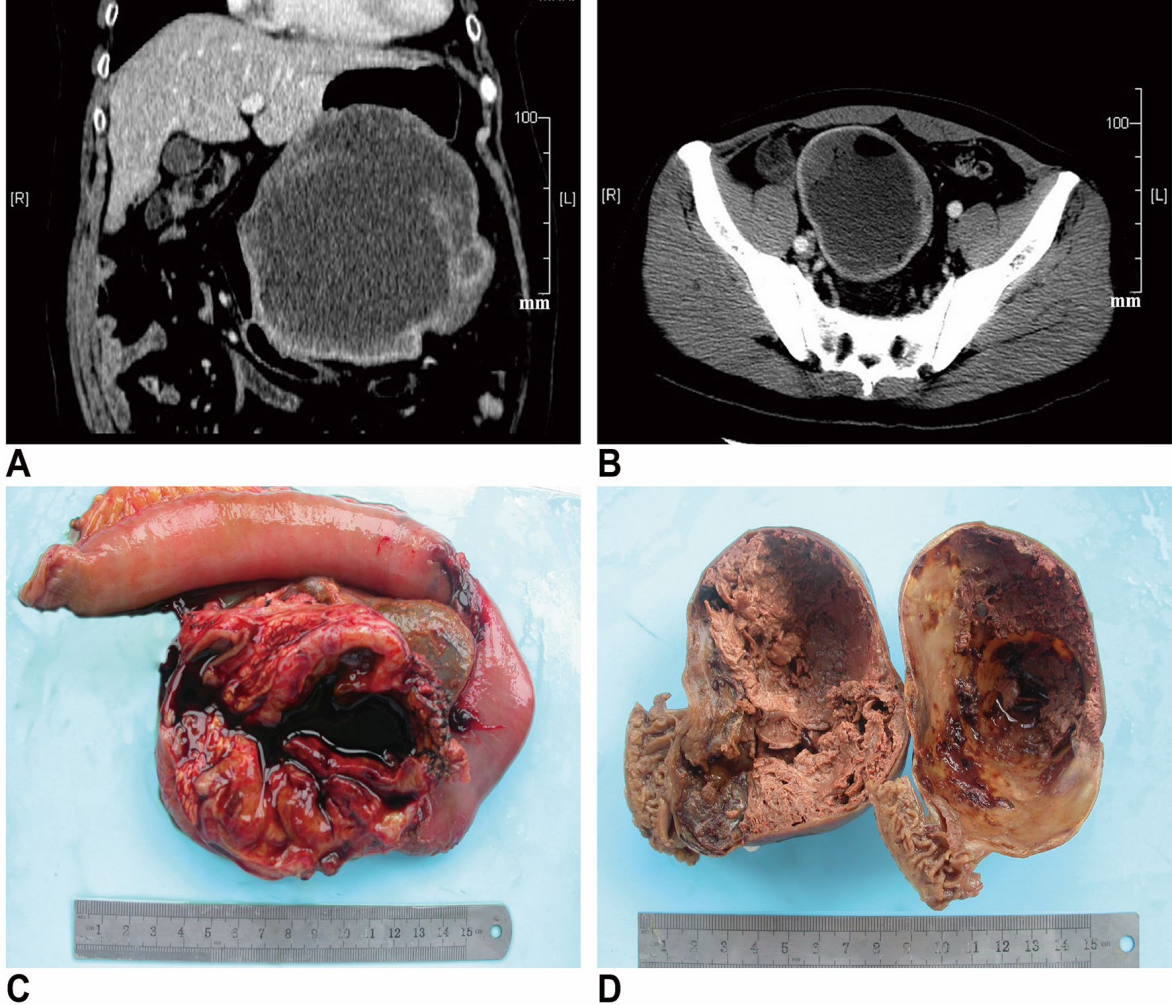
On contrast-computed tomography (CT), cGISTs usually demonstrate an exophytic, well-defined, low-density mass with peripheral enhancement (**a, b**). Grossly, the cystic component made up the vast majority of the masses and was surrounded by neoplastic parenchyma variable in thickness. Most of the cysts were filled by dark bloody serous fluid and necrotic debris (**c, d**)

Clinical and follow-up data are summarized in Table 1. Of the 20 patients included in this study, 9 were males and 11 females, with a mean age of 61 years (range 31–73 years) at diagnosis. Clinical presentations were known in 16 patients: 5 patients presented with abdominal pain or discomfort, 4 had gastrointestinal bleeding and 2 presented with abdominal mass. The remaining 5 patients were discovered incidentally by physical or imaging examinations for other reasons. The original location of cGISTs were the stomach in ten patients, the jejunum or ileum in seven, the duodenum in two and the omentum in one. Preoperative computed tomography (CT) or magnetic resonance imaging (MRI) reports were available for all patients but did not provide robust evidence for differential diagnosis with other cystic lesions. cGISTs usually demonstrated as an exophytic, well-defined, low-density mass with peripheral enhancement on contrast imaging (Fig. 1a, b). Endoscopic ultrasound (EUS) was performed only in 1 patient and showed a hypoechoic structure arising from the fourth layer of the stomach.

**Table 1:** Clinical and follow-up information of 20 cases of cGIST

Case	Age (years)/sex	Clinical presentation	Radiologic findings	Location	Treatment	Follow-up (months)	Status
1	66M	NA	Abdominal occupying lesion	Small intestine	Laparotomy	60	ANED
2	71F	Gastrointestinal bleeding	Abdominal occupying lesion	Stomach	Laparotomy	143	ANED
3	55F	Abdominal discomfort	Occupying lesion between liver and stomach	Stomach	Laparotomy	103	ANED
4	42M	NA	Abdominal occupying lesion	Small intestine	Laparotomy	128	ANED
5	50F	Gastrointestinal bleeding	Occupying lesion in the head of pancreas: GISTs?	Duodenum	Laparotomy	128	ANED
6	71F	Incidental findings during examination for cystic disease of kidney	Cystic lesion in the tail of pancreas	Stomach	Laparotomy	123	ANED
7	35F	Abdominal mass	Ovarian cyst	Small intestine	Laparotomy+ Imatinib	39	ANED
8	73F	Incidental finding during examination for appendix mucinous adenocarcinoma	Abdominal malignant tumor: GISTs?	Small intestine	Laparotomy	4	DUD
9	31F	Gastrointestinal bleeding	Abdominal occupying lesion	Stomach	Laparotomy	75	ANED
10	72F	Abdominal mass	Cystic lesion between liver and stomach: GISTs	Stomach	Laparotomy	79	ANED
11	61F	Incidental finding during physical examination	Abdominal cystic lesion: GISTs?	Stomach	Laparotomy	36	ANED
12	62M	Abdominal pain	Abdominal cystic lesion: GISTs?	Stomach	Laparotomy	63	ANED
13	58M	Abdominal discomfort	Abdominal occupying lesion	Stomach	Laparotomy	51	ANED
14	47M	Gastrointestinal bleeding	Abdominal occupying lesion	Duodenum	Laparotomy	59	AWD
15	61F	NA	Abdominal occupying lesion	Stomach	Laparotomy	36	ANED
16	68M	Abdominal discomfort	Abdominal occupying lesion	Stomach	Laparotomy + imatinib	45	ANED
17	57M	Abdominal pain	Diverticulum or GISTs	Small intestine	Laparotomy	19	ANED
18	61F	Incidental finding during physical examination	Ovarian chocolate cyst	Omentum	Laparotomy	30	ANED
19	45M	NA	Abdominal occupying lesion	Small intestine	Laparotomy	66	ANED
20	64M	Incidental finding during physical examination	Pelvic occupying lesion	Small intestine	Laparotomy + imatinib	92	ANED

*ANED* alive with no evidence of disease, *DUD* died of unrelated disease, *AWD* alive with disease

### Pathologic findings

Pathologic findings are presented in Table 2. On macroscopic examination, cGISTs appeared as soft, well-circumscribed masses and ranged in size from 7 to 20 cm with a mean of 11.5 cm (Fig. 1c, d). Most of the neoplasms were unilocular (*n* = 15, 75%). On sectioning, the cut surface varied in color from gray/white to red/brown depending on the degree of hemorrhage. The cystic component made up the vast majority of the masses and was surrounded by neoplastic parenchyma variable in thickness (range 0.1–4.5 cm). Most of the cysts (14/16, 87.5%) were filled by dark bloody fluid and necrotic debris but one contained grey-green turbid, the other light-yellow fluid.

**Table 2:** Pathologic details of 20 cases of cGIST

Case	Size (cm)	Focality	Cystic contents	Sectioning	Cellularity	Cellular type	Mitosis (/50 HPF)	Cellular atypia
1	15	Unilocular	Dark bloody necrosis debris	NA	Moderate	Epithelioid	0	Mild–moderate
2	12	Unilocular	Dark brown fluid	Grey/white	Moderate	Epithelioid	3	Mild–moderate, partially severe
3	8	Multilocular	Light yellow clear fluid	Grey/red	Moderate	Epithelioid	2	Moderate–severe
4	8	Unilocular	Dark bloody necrotic debris	Grey/white	Moderate	Spindle	0	Mild–moderate
5	10	Unilocular	Dark brown fluid	Grey/white	Moderate	Spindle	1	Mild–moderate, partially severe
6	7	Unilocular	Dark red fluid	Grey/red	Moderate	Spindle	2	Mild–moderate
7	9	Unilocular	Dark bloody necrotic debris	Grey/brown	Moderate	Spindle	2	Mild–moderate
8	8	Unilocular	Grey–green turbid fluid	Grey/red	Moderate	Spindle	1	Mild–moderate
9	8.5	Unilocular	NA	NA	Moderate-Condense	Spindle	1	Moderate
10	16	Unilocular	Grey–red turbid fluid	Grey/white	Moderate	Epithelioid	0	Moderate–severe
11	12.5	Multilocular	NA	Grey/white	Moderate	Spindle	1	Mild–moderate
12	20	Multilocular	Dark red fluid	Grey/white	Moderate	Epithelioid	0	Moderate, partially severe
13	13.5	Unilocular	Brown fluid	Brown	Moderate	Spindle	0	Mild
14	12	Unilocular	Dark red necrotic debris	Grey/white	Moderate	Spindle	2	Mild–moderate
15	13	Unilocular	NA	NA	Moderate	Epithelioid	11	Moderate
16	15	Multilocular	Dark bloody necrotic debris	Grey/white	Moderate	Spindle	1	Mild–moderate
17	9	Unilocular	Dark red necrotic debris	Grey/brown	Moderate	Spindle	0	Moderate–severe
18	8	Unilocular	NA	NA	Moderate	Spindle	8	Moderate
19	14	Unilocular	Dark bloody necrotic debris	NA	Mild	Spindle	1	Moderate
20	13	Multilocular	Dark bloody serous fluid	Grey/red	Moderate	Spindle	0	Mild–moderate

**Fig. 2:**
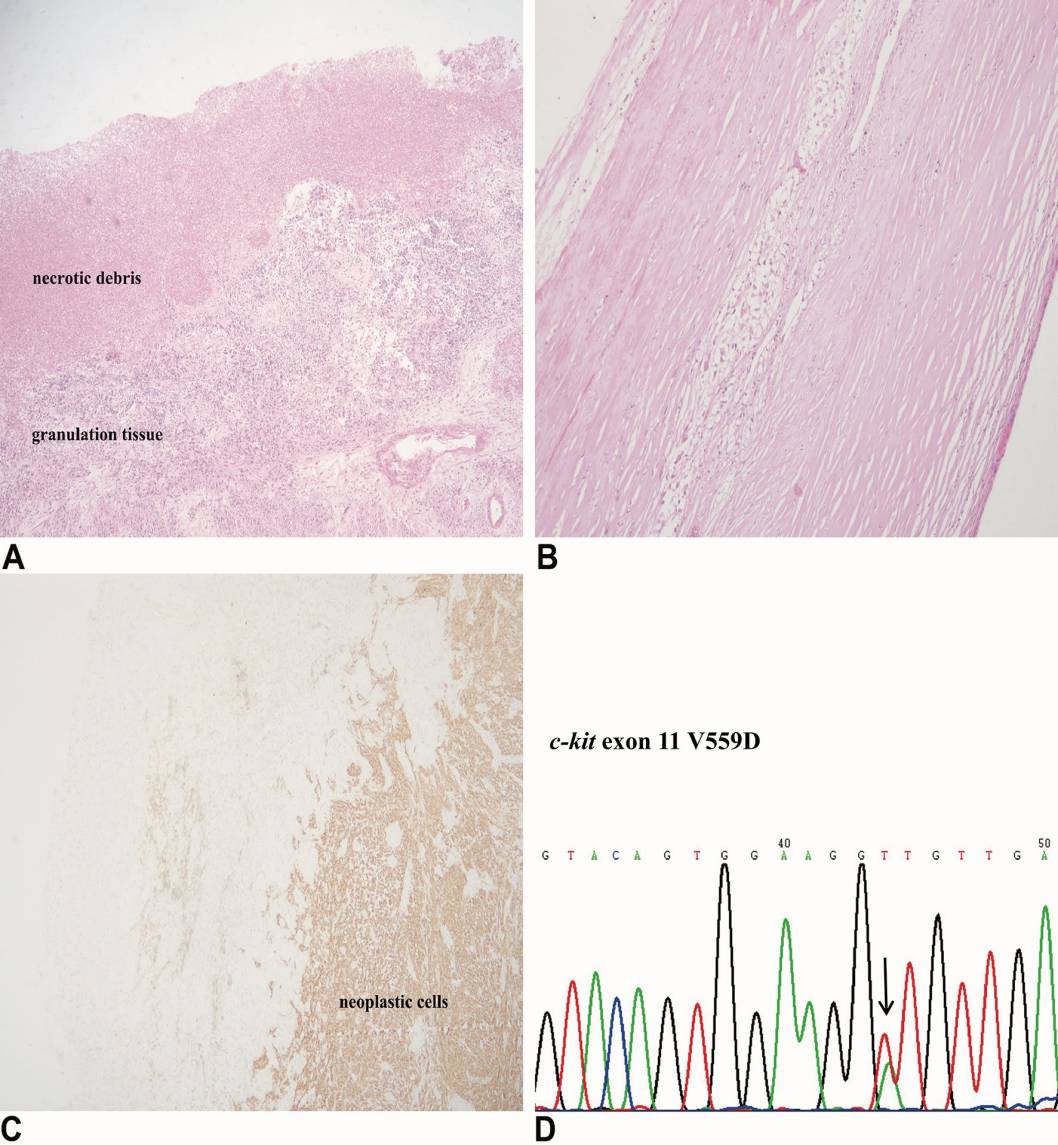
At low magnification, cyst wall was composed of neoplastic spindle/epithelioid cells and collagenous fiber lined with necrotic debris and granulation tissue (**a**). In rare cases were the cyst wall occupied by collagenous fiber with little cellular component (**b**). All available cases demonstrated positive staining for CD117 (**c**). *c-kit* exon 11 point substitution was the most common among all *c-kit*/*PDGFRα* mutants (**d**)

Microscopically, cyst wall was composed of neoplastic spindle (*n* = 14, 70%)/epithelioid cells (*n* = 6, 30%) and collagenous fiber with necrotic debris and granulation tissue lining on the inner surface (Fig. 2a). The degree of cellularity varied in different cases and areas but was moderate in general. In rare cases were the cyst wall occupied by collagenous fiber with little cellular component (Fig. 2b). The nuclei were of mild to moderate atypia in 17 (85%) patients and mitotic count was fewer than 5/50 HPF in 18 (90%) patients. In addition, hemosiderin deposits and foamy histocytes were observed in several cases. Within the cysts, there were liquefactive necrosis, hemorrhage and myxoid changes but neoplastic cells were absent. Malignant biological parameters, such as severe nuclear atypia, mucosal infiltration and muscularis propria infiltration, were discovered in nine patients but none involved more than two parameters.

The results of immunohistochemical and mutational analysis are summarized in Table 3. All cases demonstrated positive staining for CD117 (20/20) and negative staining for desmin (20/20) (Fig. 2c). DOG-1 was positive in 14/15 (93.3%) cases, CD34 was positive in 16/19 (84.2%) cases, and SMA was positive in 7/20 (35%) cases. Staining was also performed for several other antibodies, but results were not included because of the limited number of cases. Mutational analyses revealed *c-kit* or *PDGFRα* mutations in eleven cases with the remaining nine being wild type for these two mutations. Among all *c-kit*/*PDGFRα* mutants, *c-kit* exon 11 point substitution was the most common (4/11), followed by *c-kit* exon 11 deletions (2/11), combination of substitution and deletions in *c-kit* exon 11 (2/11), PDGFR*α* exon 18 D842V substitution (2/11) and c-kit exon 9 deletions (1/11).

**Table 3:** Immunohistochemical and mutational analysis of 20 case of cGIST

Case	Malignant parameters	Malignancy	NIH criterion	CD117	Desmin	DOG-1	CD34	SMA	S-100	Mutational status
1	No	Nonmalignant	High	Pos	Neg	NA	Pos	Pos	NA	None in *c-kit* or *PDGFRα*
2	Severe nuclear atypia	Low	High	Pos	Neg	Pos	Neg	Pos	Neg	None in *c-kit* or *PDGFRα*
3	Infiltration of muscularis propria and vessels	Low	Intermediate	Pos	Neg	Pos	Pos	Neg	Pos	None in *c-kit* or *PDGFRα*
4	No	Nonmalignant	High	Pos	Neg	Pos	Pos	Neg	NA	None in *c-kit* or *PDGFRα*
5	Severe nuclear atypia	Low	High	Pos	Neg	Pos	Pos	Pos	Pos	*c-kit* exon 11 Y568S, DEL569-573
6	No	Nonmalignant	Intermediate	Pos	Neg	Pos	Pos	Neg	Neg	*c-kit* exon 11 V559D
7	No	Nonmalignant	High	Pos	Neg	Pos	Pos	Pos	Neg	None in *c-kit* or *PDGFRα*
8	Muscularis propria infiltration	Low	High	Pos	Neg	Pos	Pos	Pos	Pos	*c-kit* exon 11 L576P
9	No	Nonmalignant	Intermediate	Pos	Neg	NA	Pos	Neg	Neg	*c-kit* exon 11 DEL559-561
10	Severe nuclear atypia	Low	High	Pos	Neg	Pos	Pos	Neg	NA	*c-kit* exon 11 V559D
11	No	Nonmalignant	High	Pos	Neg	Pos	Pos	Neg	NA	*c-kit* exon 11 DEL557-558
12	Severe nuclear atypia	Low	High	Pos	Neg	Pos	Pos	Neg	Neg	*PDGFRα* exon 18 D842V
13	No	Nonmalignant	High	Pos	Neg	Neg	Pos	Neg	NA	*PDGFRα* exon 18 D842V
14	Infiltration of mucosa and muscularis propria	Low	High	Pos	Neg	NA	Pos	Pos	Neg	*c-kit* exon 11 Y553D, DEL554-572
15	No	Nonmalignant	High	Pos	Neg	Pos	NA	Neg	NA	None in *c-kit* or *PDGFRα*
16	No	Nonmalignant	High	Pos	Neg	Pos	Pos	Neg	Neg	*c-kit* exon 11 V559D
17	Severe nuclear atypia	Low	High	Pos	Neg	Pos	Pos	Neg	Neg	*c-kit* exon 9 INS502-503
18	Severe nuclear atypia	Low	High	Pos	Neg	Pos	Pos	Neg	Neg	None in *c-kit* or *PDGFRα*
19	No	Nonmalignant	High	Pos	Neg	NA	Neg	Pos	NA	None in *c-kit* or *PDGFRα*
20	No	Nonmalignant	High	Pos	Neg	NA	Neg	Neg	Neg	None in *c-kit* or *PDGFRα*

*DEL* deletion, *INS* insertion

### Treatment and follow-up

All patients underwent laparotomy for surgical resection of cGISTs and no severe complication occurred postoperatively. According to modified National Institute of Health (NIH) criterion, the risk of recurrence was estimated to be intermediate in 3 cases (15%) and high in 17 cases (85%). Based on biological behaviors proposed for evaluating GISTs by our institute, 11 patients were classified as nonmalignant and 9 as low degree of malignancy (Table 3). Adjuvant imatinib was carried out in 3 nonmalignant/high-risk patients (12, 30 and 39 months respectively) and no evidence of recurrence was observed during follow-up (45, 92 and 39 months respectively). For patients without adjuvant imatinib, local recurrence was detected in one (low degree of malignancy/high risk) after a median duration of 63 months (range 4–143 months). The patient underwent a second operation and postoperative imatinib therapy and was progression free after another follow-up of 35 months. One patient died of an unrelated cause 4 months after surgery, whereas all the other patients were alive till the end of follow-up (Table 1).

### Comparison between cystic and solid GISTs

**Fig. 3:**
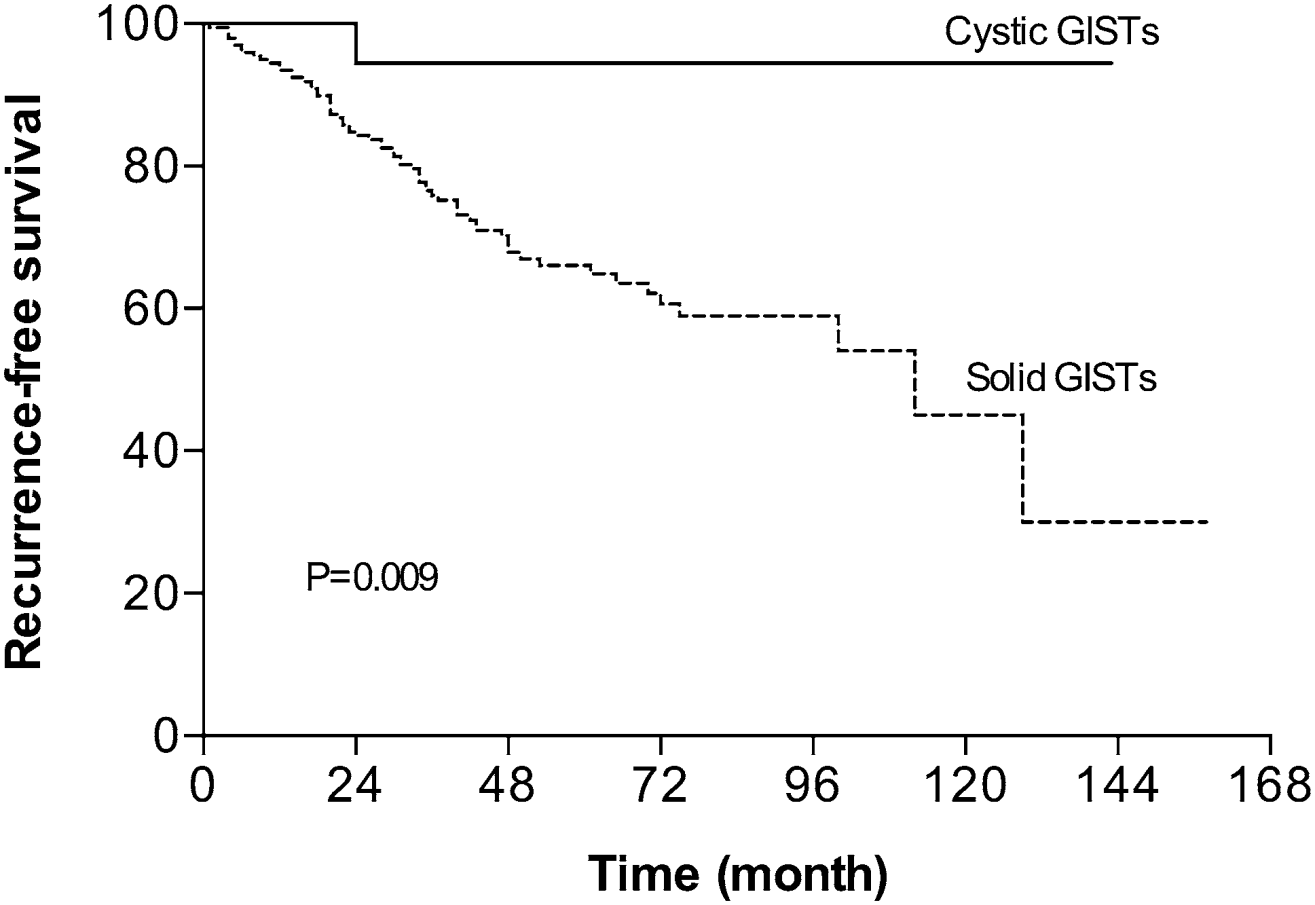
Recurrence-free survival analysis (RFS) of 20 cystic and 200 solid GISTs

The clinicopathological characteristics of cystic GISTs in comparison with their solid counterparts are summarized in Table 4. There was no difference between the two groups in terms of age, sex, tumor location, cellular type, NIH criterion and adjuvant imatinib, except for mitotic index and numbers of biological parameters. Eighteen (90%) cGISTs had a mitotic rate of five or fewer per 50 HPF with a median of 1/50 HPF. The number of malignant biological parameters was none in 9 (45%) cGISTs and 1–2 in 11 (55%). In comparison, 85 of the 200 (42.5%) solid GISTs had mitoses more than 10/50 HPF and 27 (13.5%) had 6 to 10 mitoses per 50 HPF. Solid GISTs were more likely to manifest malignant biological behaviors, with more than 2 parameters in 81 (40.5%) cases and 1–2 parameters in 77 (38.5%) cases. Of 167 solid GISTs with mutational information, 103 (61.7%) harbored mutations in *c-kit* exon 11, 41 (24.5%) in *c-kit* exon 9, 1 (0.6%) in *c-kit* exon 13, 4 (2.4%) in *PDGFRα* exon 18 D842V and 18 (10.8%) in none. In addition, survival analysis showed that patients with solid GISTs had a significantly worse recurrence-free survival (5-year RFS = 66.1%) than patients with cGISTs (5-year RFS = 94.4%) (Fig. 3).

**Table 4:** Comparison of clinicopathological characteristics between cystic and solid GISTs

	Cystic (*n* = 20)	Solid (*n* = 200)	*P*
Age (years)	59.5 (66–73)	60 (19–84)	0.554
Sex			
Male	9	116	0.263
Female	11	84	
Location			
Stomach	10	117	0.462
Non-stomach	10	83	
Cellular type			
Spindle	14	175	0.071
Non-spindle	6	25	
Size (cm)	9 (7–20)	12 (7–20)	0.056
7–9.9	8	102	0.601
10–14.9	8	70	
≥ 15	4	28	
Mitotic Index (/50 HPF)	1 (0–11)	8 (1–210)	< 0.001*
≤ 5	18	88	0.0004*
6–10	1	27	
> 10	1	85	
NIH criterion			
Intermediate	3	32	1
High	17	168	
Predictive parameters of malignancy	0 (0–2)	2 (1–6)	< 0.001*
0	11	42	< 0.001*
1–2	9	77	
> 2	0	81	
Mutational status			
*c-kit*	9	145	< 0.001*
*PDGFRα*	2	4	
Neither	9	18	
Adjuvant imatinib			
Yes	3	49	0.498
No	17	151	

*P < 0.05 was considered statistically significant

## Discussion

Gastrointestinal stromal tumors typically appear as regular, soft, solid masses, varying greatly in size and distributed within and outside the gastrointestinal tract. They are usually well circumscribed and unencapsulated with an endophytic or exophytic growth pattern. Small cystic areas are frequently observed in GISTs with large size, but GISTs rarely manifest predominantly as cystic tumors. Cases of this uncommon form have been described but no retrospective study has been reported in the English literature (Hamza et al. 2016; Okano et al. 2015; Shaikh et al. 2015; Sun et al. 2016; Takahashi et al. 2010; Wang et al. 2017; Zhu et al. 2014). In addition, clinical follow-up information was not available in most cases. In the present study, a cohort of 20 GISTs with extensive cystic change, designated as cGISTs, were evaluated to characterize their clinicopathological features and determine their biological behaviors and prognoses.

Most of the cGISTs, both in our study and prior reports, demonstrated an exophytic growth pattern and lacked pathognomonic signs or symptoms until the late stage of the disease. CT or MRI played an important role in differential diagnosis, providing evidence for original locations while excluding inconsistent neoplasms, but was not potent enough to establish exact diagnosis preoperatively. Although suggested as a useful modality for diagnosing GISTs, EUS-guided FNA appears as a prudent choice for cGISTs due to the possibility of insufficient sample volume and fear of dissemination. For all these reasons, misdiagnoses were easily made preoperatively, which included duplication cysts, mucin-producing tumors, pancreatic pseudocysts, cystic lymphangioma, cystic degeneration of other solid neoplasms, etc. Therefore, successful diagnosis of GISTs necessitates further histological and immunohistochemical examinations. Of note, diagnosis of GISTs should be considered when cystic tumors of unknown origin are encountered in the abdomen.

Pathological analysis of cGISTs revealed similar morphologic and immunohistochemical features with solid ones except that cGISTs were less likely to demonstrate malignant biological behaviors. cGISTs usually compressed or dislocated rather than invaded abutting organs. On microscopy, the mitotic figures were fewer than 5/50 HPF in the majority and malignant parameters such as mucosal invasion and muscularis propria infiltration were less common compared to solid GISTs of similar size (Table 3). Prominent cystic change may be responsible for their indolent behaviors, leaving only a small proportion of viable tumor cells. Cystic change of GISTs takes place in the following situations: (a) primary GISTs with expansive growth pattern, in which cystic structure takes up a large proportion, (b) cystic change induced by rapid growth rate and subsequent necrosis in malignant GISTs, (c) metastatic lesions to liver and pancreas which is cystic in nature, (d) GISTs on treatment with imatinib (Bechtold et al. 2003). Different from cystic change caused by rapid tumor growth, cGISTs are characterized by a relatively even cyst wall and fewer parameters of malignancy. As for their low frequency of *c-kit*/*PDGFRα* mutations, lack of enough neoplastic cells may be an explanation, or it is a unique inherent feature associated with their development.

To date, little is known about the cause of predominant cystic change in GISTs and we speculate that exophytic growth pattern with a small area of attachment may restrict blood supply to the tumor. Aggravated by occasional vascular obstruction and incapability of angiogenesis, congestion, hemorrhage, degeneration and liquefactive necrosis occur, resulting in remarkable cystic change. Alternatively, cystic development may be attributed to communication between tumor mass and gastrointestinal tract in certain cases. In three previously reported cases as well as one included in the present study, it is likely that ulceration of gastrointestinal mucosa allows entrance of enteric leakage into tumor mass and induces abscess formation subsequently.

Patients with cGISTs reported in the literature usually underwent surgical resection as the primary treatment but follow-up information was available in fewer than half. In our series, surgery was also the therapy of choice, with three patients receiving additional adjuvant imatinib. Recurrence was detected in one patient without adjuvant imatinib (low degree of malignancy/high risk) and brought under control by surgery and imatinb. In comparison, the 5-year RFS was 66.1% in patients with solid GISTs of similar size. We can say from our experience that surgery is safe and effective for patients with cGISTs. In view of lack of pathology and risk of rupture, preoperative administration of imatinib is not warranted. According to modified NIH criterion, the majority of cGISTs (85% in our series) was stratified as high risk of recurrence and necessitates adjuvant imatinib. However, to avoid excessive administration of imatinib, we recommend meticulous evaluation of malignant parameters prior to decision. For patients with cGISTs classified as nonmalignant, surgical resection alone may achieve long-term recurrence free survival; for patients with cGISTs classified as low degree of malignancy, adjuvant imatinib should be considered but its benefit might be counteracted by low incidence of recurrence.

Several limitations were implicit in our study. First, the cases included were from surgical pathology database and consultation files over a long period of time, resulting in incomplete clinical and pathological information. Second, it was a retrospective study with a limited sample size, which renders the conclusions provisional and warrants further investigations. Third, the magnitude of cystic change was assessed according to radiology reports and/or gross reports. A more qualitative determination of tumor cells may need to perform during radiologic and pathological examinations.

In conclusion, although similar to solid GISTs in terms of morphologic and immunohistochemical features, cGISTs should be considered as a specific subtype of GISTs with relatively indolent behaviors and favorable prognoses. Parameters of malignancy are more applicable than modified NIH criterion in determining recurrence risk and whether to administrate adjuvant imatinib. Future studies analyzing a larger cohort with more detailed information should help shed light on the pathogenesis and long-term survival for these neoplasms.
